# Crystal structure of 2-(3-bromo­phen­yl)-1,3-di­thiane

**DOI:** 10.1107/S2056989015002832

**Published:** 2015-02-13

**Authors:** Julio Zukerman-Schpector, Ignez Caracelli, Hélio A. Stefani, Olga Gozhina, Edward R. T. Tiekink

**Affiliations:** a Departmento de Química, Universidade Federal de São Carlos, 13565-905 São Carlos, SP, Brazil; b Departmento de Física, Universidade Federal de São Carlos, 13565-905 São Carlos, SP, Brazil; c Departamento de Farmácia, Faculdade de Ciências Farmacêuticas, Universidade de São Paulo, 05508-900 São Paulo-SP, Brazil; d Department of Chemistry, University of Malaya, 50603 Kuala Lumpur, Malaysia

**Keywords:** crystal structure, 1,3-di­thiane, conformation, C—H⋯π inter­actions, π–π inter­actions

## Abstract

In the title compound, C_10_H_11_BrS_2_, the 1,3-di­thiane ring has a chair conformation with the 1,4-disposed C atoms being above and below the remaining four atoms. The bromo­benzene ring occupies an equatorial position and forms a dihedral angle of 86.38 (12)° with the least-squares plane through the 1,3-di­thiane ring. Thus, to a first approximation the mol­ecule has mirror symmetry with the mirror containing the bromo­benzene ring and the 1,4-disposed C atoms of the 1,3-di­thiane ring. In the crystal, mol­ecules associate *via* weak methyl­ene–bromo­benzene C—H⋯π and π–π [*Cg*⋯*Cg* = 3.7770 (14) Å for centrosymmetrically related bromo­benzene rings] inter­actions, forming supra­molecular layers parallel to [10-1]; these stack with no specific inter­molecular inter­actions between them.

## Related literature   

For the original synthesis and characterization of the title compound, see: Ballesteros *et al.* (2005 ▸). For the structure of the unsubstituted parent compound which is virtually superimposable on the title compound, see: Kalff & Romers (1966 ▸).
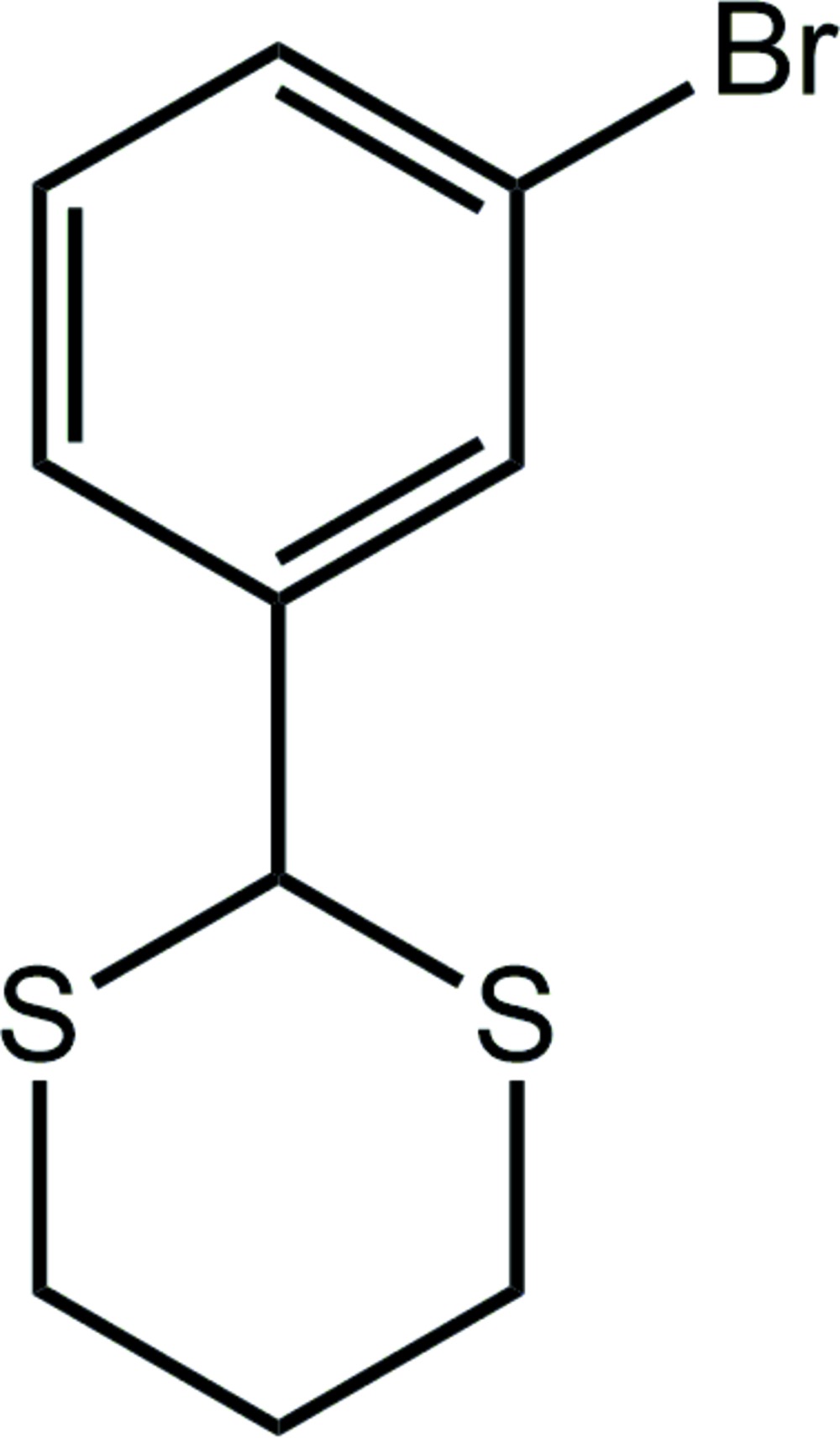


## Experimental   

### Crystal data  

C_10_H_11_BrS_2_
*M* *_r_* = 275.22
Monoclinic, 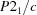

*a* = 8.9821 (4) Å
*b* = 11.3871 (5) Å
*c* = 11.0550 (5) Å
β = 99.604 (3)°
*V* = 1114.86 (9) Å^3^
*Z* = 4
Mo *K*α radiation
μ = 4.01 mm^−1^
*T* = 296 K
0.33 × 0.28 × 0.16 mm

### Data collection  

Bruker APEXII CCD diffractometer
Absorption correction: multi-scan (*SADABS*; Sheldrick, 1996 ▸) *T*_min_ = 0.374, *T*_max_ = 0.745
7307 measured reflections
2060 independent reflections
1820 reflections with *I* > 2σ(*I*)
*R*_int_ = 0.029

### Refinement  

*R*[*F* ^2^ > 2σ(*F* ^2^)] = 0.031
*wR*(*F* ^2^) = 0.082
*S* = 1.06
2060 reflections
118 parameters
H-atom parameters constrained
Δρ_max_ = 0.54 e Å^−3^
Δρ_min_ = −0.65 e Å^−3^

### 

Data collection: *APEX2* (Bruker, 2009 ▸); cell refinement: *SAINT* (Bruker, 2009 ▸); data reduction: *SAINT*; program(s) used to solve structure: *SIR2014* (Burla *et al.*, 2015 ▸); program(s) used to refine structure: *SHELXL2014* (Sheldrick, 2015 ▸); molecular graphics: *ORTEP-3 for Windows* (Farrugia, 2012 ▸) and *DIAMOND* (Brandenburg, 2006 ▸); software used to prepare material for publication: *MarvinSketch* (ChemAxon, 2010 ▸) and *publCIF* (Westrip, 2010 ▸).

## Supplementary Material

Crystal structure: contains datablock(s) I, New_Global_Publ_Block. DOI: 10.1107/S2056989015002832/hg5429sup1.cif

Structure factors: contains datablock(s) I. DOI: 10.1107/S2056989015002832/hg5429Isup2.hkl

Supporting information file. DOI: 10.1107/S2056989015002832/hg5429Isup3.cml

**Fig. 1.**
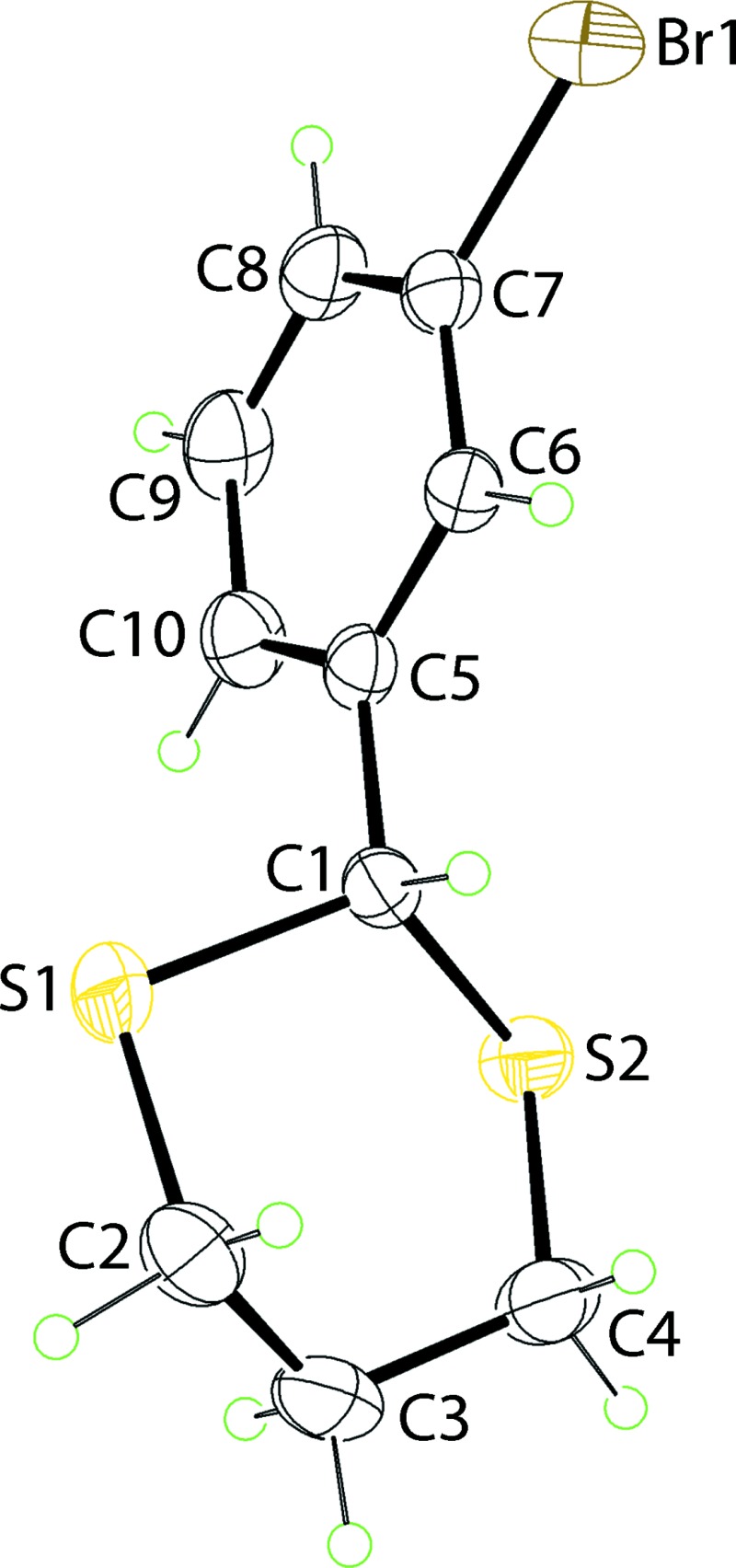
The molecular structure of the title compound showing the atom-labelling scheme and displacement ellipsoids at the 35% probability level.

. DOI: 10.1107/S2056989015002832/hg5429fig1.tif
The mol­ecular structure of the title compound showing the atom-labelling scheme and displacement ellipsoids at the 35% probability level.

**Fig. 2.**
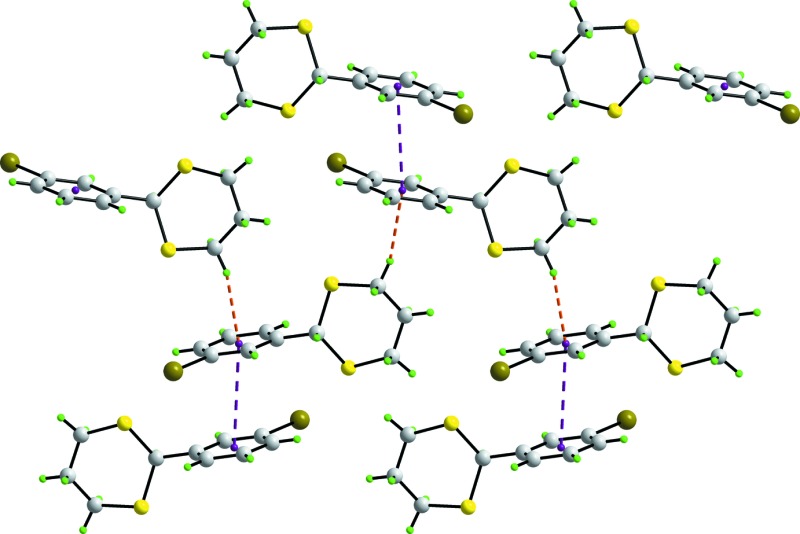
A view of the supramolecular layer parallel to [101] mediated by C—H···π and π—π interactions shown as orange and purple dashed lines, respectively.

**Fig. 3.**
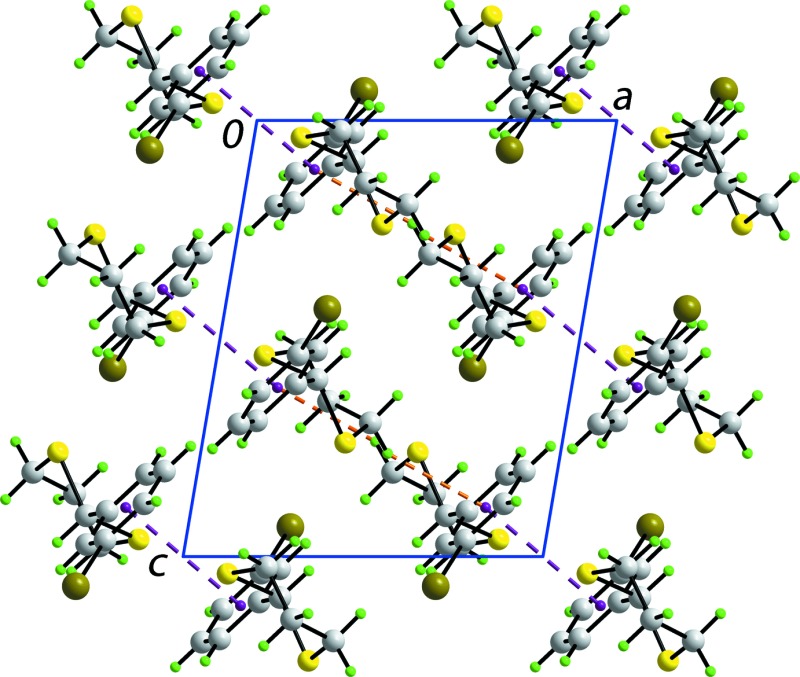
A view in projection down the b axis of the unit-cell contents. The C—H···π and π—π interactions shown as orange and purple dashed lines, respectively.

. DOI: 10.1107/S2056989015002832/hg5429fig2.tif
A view of the supra­molecular layer parallel to [10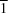] mediated by C—H⋯π and π—π inter­actions shown as orange and purple dashed lines, respectively.

b . DOI: 10.1107/S2056989015002832/hg5429fig3.tif
A view in projection down the *b* axis of the unit-cell contents. The C—H⋯π and π—π inter­actions shown as orange and purple dashed lines, respectively.

CCDC reference: 1048592

Additional supporting information:  crystallographic information; 3D view; checkCIF report

## Figures and Tables

**Table 1:** Hydrogen-bond geometry (, ) *Cg*1 is the centroid of the C5C10 ring.

*D*H*A*	*D*H	H*A*	*D* *A*	*D*H*A*
C2H2b*Cg*1^i^	0.97	2.83	3.668(4)	146

Symmetry code: (i) 
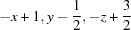
.

## supplementary crystallographic information

### S1. Experimental

A solution of 3-bromobenzaldehyde (0.037 mol, 1 equiv.) in chloroform (20 ml) was combined with an equimolar amount of propane-1,3-dithiol (3.7 ml, 0.037 mol) at room temperature. The solution was stirred for 1 h at this temperature, then cooled to -20 °C after which BF_3_ etherate (0.46 ml, 0.0037 mol, 0.1 equiv.) was added drop-wise. The reaction solution was allowed to warm to room temperature and stirred overnight. After this time, the solution was washed three times each with water, 10% aqueous KOH, then water followed by drying over MgSO_4_. Evaporation of the solvent furnishes a pure product as colourless crystals in 90% yield. To obtain crystals suitable for X-ray analysis, the product was crystallized from CH_3_OH. The spectroscopic data matched those reported in the literature (Ballesteros *et al.*, 2005).

### S2. Refinement

Carbon-bound H-atoms were placed in calculated positions (C—H = 0.95–0.98 Å) and were included in the refinement in the riding model approximation, with *U*_iso_(H) = 1.2 *U*_eq_(C).

### Crystal data

**Table:**

C_10_H_11_BrS_2_	*F*(000) = 552
*M**_r_* = 275.22	*D*_x_ = 1.640 Mg m^−^^3^
Monoclinic, *P*2_1_/*c*	Mo *K*α radiation, λ = 0.71073 Å
*a* = 8.9821 (4) Å	Cell parameters from 4286 reflections
*b* = 11.3871 (5) Å	θ = 2.6–25.4°
*c* = 11.0550 (5) Å	µ = 4.01 mm^−^^1^
β = 99.604 (3)°	*T* = 296 K
*V* = 1114.86 (9) Å^3^	Prism, colourless
*Z* = 4	0.33 × 0.28 × 0.16 mm

### Data collection

**Table:**

Bruker APEXII CCD diffractometer	1820 reflections with *I* > 2σ(*I*)
φ and ω scans	*R*_int_ = 0.029
Absorption correction: multi-scan (*SADABS*; Sheldrick, 1996)	θ_max_ = 25.4°, θ_min_ = 2.3°
*T*_min_ = 0.374, *T*_max_ = 0.745	*h* = −9→10
7307 measured reflections	*k* = −13→12
2060 independent reflections	*l* = −13→13

### Refinement

**Table:**

Refinement on *F*^2^	0 restraints
Least-squares matrix: full	Hydrogen site location: inferred from neighbouring sites
*R*[*F*^2^ > 2σ(*F*^2^)] = 0.031	H-atom parameters constrained
*wR*(*F*^2^) = 0.082	*w* = 1/[σ^2^(*F*_o_^2^) + (0.0384*P*)^2^ + 0.6522*P*] where *P* = (*F*_o_^2^ + 2*F*_c_^2^)/3
*S* = 1.06	(Δ/σ)_max_ = 0.002
2060 reflections	Δρ_max_ = 0.54 e Å^−^^3^
118 parameters	Δρ_min_ = −0.65 e Å^−^^3^

### Special details

**Table:**

Geometry. All e.s.d.'s (except the e.s.d. in the dihedral angle between two l.s. planes) are estimated using the full covariance matrix. The cell e.s.d.'s are taken into account individually in the estimation of e.s.d.'s in distances, angles and torsion angles; correlations between e.s.d.'s in cell parameters are only used when they are defined by crystal symmetry. An approximate (isotropic) treatment of cell e.s.d.'s is used for estimating e.s.d.'s involving l.s. planes.

### Fractional atomic coordinates and isotropic or equivalent isotropic displacement parameters (Å^2^)

**Table:**

	*x*	*y*	*z*	*U*_iso_*/*U*_eq_	
Br1	0.71577 (4)	1.19362 (3)	1.06788 (3)	0.06773 (15)	
S1	0.60308 (9)	0.70880 (7)	0.76097 (6)	0.0565 (2)	
S2	0.86833 (7)	0.65738 (6)	0.96061 (6)	0.04880 (19)	
C1	0.7115 (3)	0.7543 (2)	0.9063 (2)	0.0373 (5)	
H1	0.6439	0.7542	0.9674	0.045*	
C2	0.5426 (4)	0.5670 (3)	0.8068 (3)	0.0657 (8)	
H2A	0.4799	0.5784	0.8692	0.079*	
H2B	0.4805	0.5306	0.7366	0.079*	
C3	0.6689 (4)	0.4839 (3)	0.8560 (3)	0.0649 (8)	
H3A	0.6262	0.4076	0.8690	0.078*	
H3B	0.7342	0.4746	0.7950	0.078*	
C4	0.7631 (3)	0.5247 (3)	0.9752 (3)	0.0587 (7)	
H4A	0.8331	0.4628	1.0065	0.070*	
H4B	0.6969	0.5378	1.0349	0.070*	
C5	0.7677 (2)	0.8778 (2)	0.8960 (2)	0.0377 (5)	
C6	0.7265 (3)	0.9638 (2)	0.9723 (2)	0.0382 (5)	
H6	0.6661	0.9450	1.0301	0.046*	
C7	0.7754 (3)	1.0778 (2)	0.9623 (2)	0.0432 (6)	
C8	0.8665 (3)	1.1081 (3)	0.8788 (2)	0.0526 (7)	
H8	0.8994	1.1851	0.8733	0.063*	
C9	0.9078 (3)	1.0224 (3)	0.8039 (2)	0.0575 (7)	
H9	0.9692	1.0417	0.7469	0.069*	
C10	0.8598 (3)	0.9074 (3)	0.8115 (2)	0.0496 (6)	
H10	0.8892	0.8503	0.7602	0.060*	

### Atomic displacement parameters (Å^2^)

**Table:**

	*U*^11^	*U*^22^	*U*^33^	*U*^12^	*U*^13^	*U*^23^
Br1	0.0883 (3)	0.0415 (2)	0.0730 (2)	0.00622 (14)	0.01259 (18)	−0.00728 (13)
S1	0.0631 (5)	0.0554 (4)	0.0434 (4)	−0.0065 (3)	−0.0132 (3)	0.0005 (3)
S2	0.0406 (3)	0.0434 (4)	0.0579 (4)	0.0002 (3)	−0.0047 (3)	0.0017 (3)
C1	0.0393 (12)	0.0381 (13)	0.0343 (12)	0.0000 (10)	0.0053 (9)	−0.0026 (10)
C2	0.0624 (18)	0.0590 (19)	0.0669 (18)	−0.0188 (15)	−0.0145 (15)	−0.0067 (15)
C3	0.075 (2)	0.0415 (16)	0.073 (2)	−0.0107 (14)	−0.0016 (16)	−0.0100 (14)
C4	0.0611 (18)	0.0409 (15)	0.0682 (18)	−0.0039 (13)	−0.0062 (14)	0.0077 (13)
C5	0.0353 (12)	0.0419 (13)	0.0339 (11)	−0.0019 (10)	−0.0002 (9)	0.0037 (10)
C6	0.0390 (12)	0.0402 (13)	0.0348 (11)	−0.0006 (10)	0.0043 (9)	0.0028 (10)
C7	0.0449 (14)	0.0395 (13)	0.0412 (13)	−0.0015 (10)	−0.0041 (10)	0.0014 (10)
C8	0.0559 (16)	0.0479 (16)	0.0507 (15)	−0.0157 (13)	−0.0007 (12)	0.0100 (12)
C9	0.0554 (17)	0.072 (2)	0.0476 (15)	−0.0166 (14)	0.0153 (13)	0.0098 (14)
C10	0.0502 (15)	0.0592 (17)	0.0414 (13)	−0.0046 (13)	0.0130 (11)	−0.0015 (12)

### Geometric parameters (Å, º)

**Table:**

Br1—C7	1.897 (3)	C4—H4A	0.9700
S1—C2	1.803 (3)	C4—H4B	0.9700
S1—C1	1.810 (2)	C5—C6	1.383 (3)
S2—C4	1.803 (3)	C5—C10	1.389 (4)
S2—C1	1.811 (2)	C6—C7	1.381 (3)
C1—C5	1.505 (3)	C6—H6	0.9300
C1—H1	0.9800	C7—C8	1.375 (4)
C2—C3	1.507 (4)	C8—C9	1.371 (4)
C2—H2A	0.9700	C8—H8	0.9300
C2—H2B	0.9700	C9—C10	1.385 (4)
C3—C4	1.515 (4)	C9—H9	0.9300
C3—H3A	0.9700	C10—H10	0.9300
C3—H3B	0.9700		
			
C2—S1—C1	98.61 (12)	S2—C4—H4A	108.8
C4—S2—C1	98.59 (13)	C3—C4—H4B	108.8
C5—C1—S1	109.74 (15)	S2—C4—H4B	108.8
C5—C1—S2	110.01 (16)	H4A—C4—H4B	107.7
S1—C1—S2	113.16 (13)	C6—C5—C10	119.2 (2)
C5—C1—H1	107.9	C6—C5—C1	119.3 (2)
S1—C1—H1	107.9	C10—C5—C1	121.5 (2)
S2—C1—H1	107.9	C7—C6—C5	119.7 (2)
C3—C2—S1	114.8 (2)	C7—C6—H6	120.1
C3—C2—H2A	108.6	C5—C6—H6	120.1
S1—C2—H2A	108.6	C8—C7—C6	121.5 (3)
C3—C2—H2B	108.6	C8—C7—Br1	120.0 (2)
S1—C2—H2B	108.6	C6—C7—Br1	118.53 (19)
H2A—C2—H2B	107.5	C9—C8—C7	118.6 (3)
C4—C3—C2	113.5 (3)	C9—C8—H8	120.7
C4—C3—H3A	108.9	C7—C8—H8	120.7
C2—C3—H3A	108.9	C8—C9—C10	121.2 (3)
C4—C3—H3B	108.9	C8—C9—H9	119.4
C2—C3—H3B	108.9	C10—C9—H9	119.4
H3A—C3—H3B	107.7	C9—C10—C5	119.8 (3)
C3—C4—S2	113.8 (2)	C9—C10—H10	120.1
C3—C4—H4A	108.8	C5—C10—H10	120.1
			
C2—S1—C1—C5	−175.91 (19)	S2—C1—C5—C10	66.0 (3)
C2—S1—C1—S2	60.79 (17)	C10—C5—C6—C7	0.9 (3)
C4—S2—C1—C5	175.20 (17)	C1—C5—C6—C7	−179.0 (2)
C4—S2—C1—S1	−61.65 (17)	C5—C6—C7—C8	−0.9 (4)
C1—S1—C2—C3	−58.8 (3)	C5—C6—C7—Br1	179.66 (17)
S1—C2—C3—C4	65.2 (4)	C6—C7—C8—C9	0.5 (4)
C2—C3—C4—S2	−65.7 (4)	Br1—C7—C8—C9	179.9 (2)
C1—S2—C4—C3	60.0 (3)	C7—C8—C9—C10	−0.2 (4)
S1—C1—C5—C6	120.8 (2)	C8—C9—C10—C5	0.3 (4)
S2—C1—C5—C6	−114.1 (2)	C6—C5—C10—C9	−0.7 (4)
S1—C1—C5—C10	−59.1 (3)	C1—C5—C10—C9	179.3 (2)

### Hydrogen-bond geometry (Å, º)

Cg1 is the centroid of the C5–C10 ring.

**Table:**

*D*—H···*A*	*D*—H	H···*A*	*D*···*A*	*D*—H···*A*
C2—H2b···*Cg*1^i^	0.97	2.83	3.668 (4)	146

Symmetry code: (i) −*x*+1, *y*−1/2, −*z*+3/2.
