# Supplementary figures and images for: Crystal structure of 2-(3-bromo­phen­yl)-1,3-di­thiane
Source: Acta Crystallogr E Crystallogr Commun. 2015 Feb 13;71(Pt 3):o179–80. doi: 10.1107/S2056989015002832 (PMC4350742; doi:10.1107/S2056989015002832)

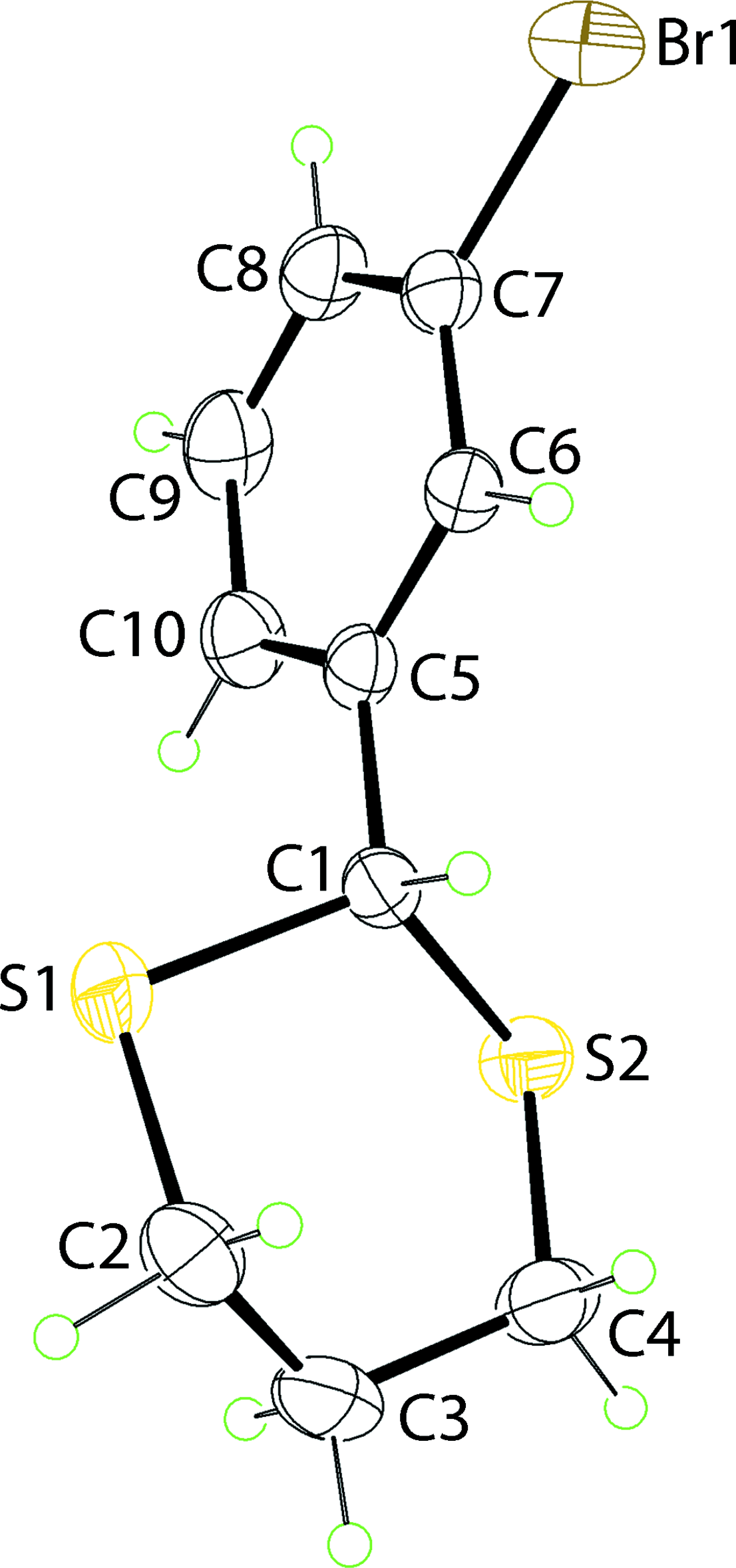

Supplement: Supplementary file 4 [file e-71-0o179-fig1.tif]

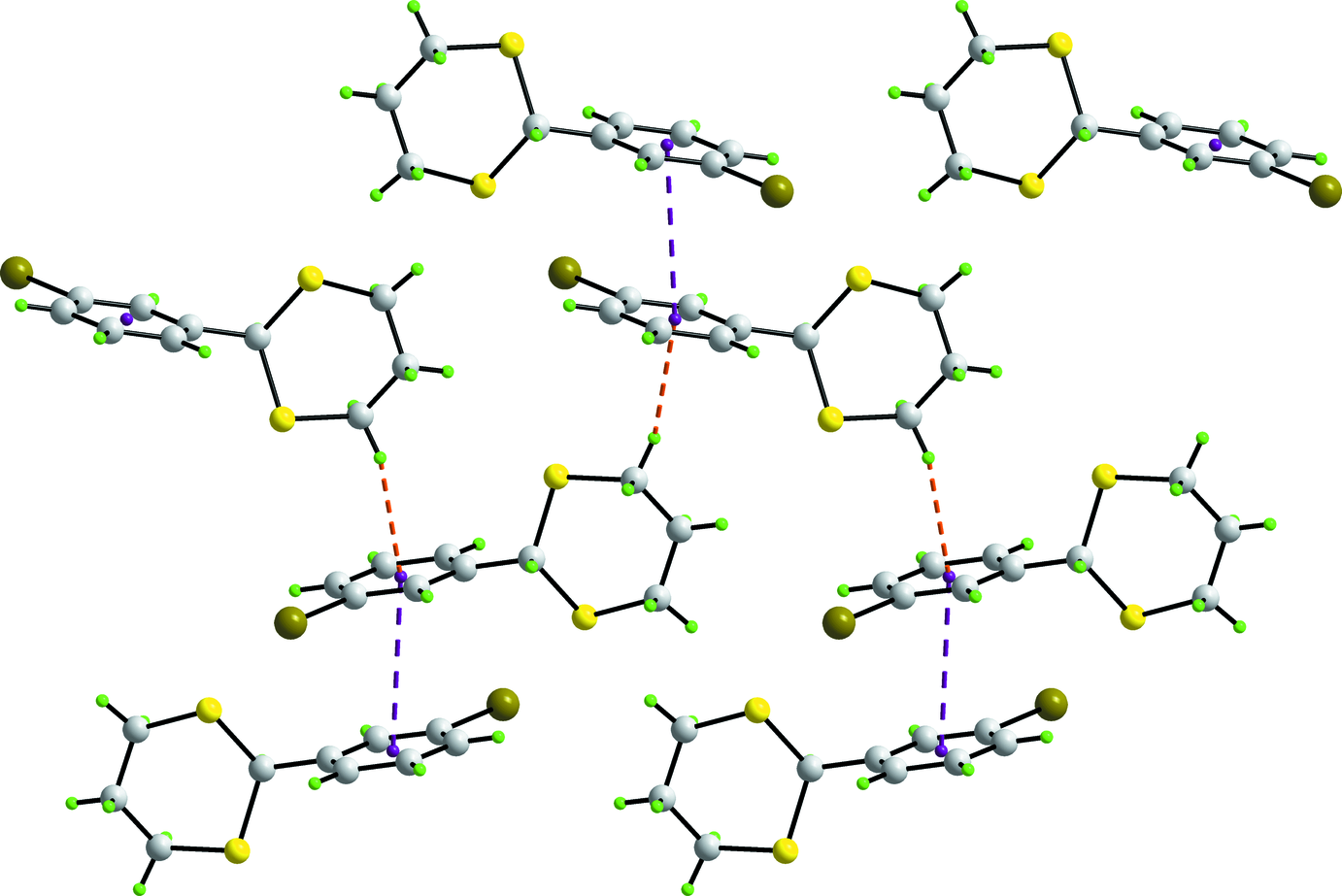

Supplement: Supplementary file 5 [file e-71-0o179-fig2.tif]

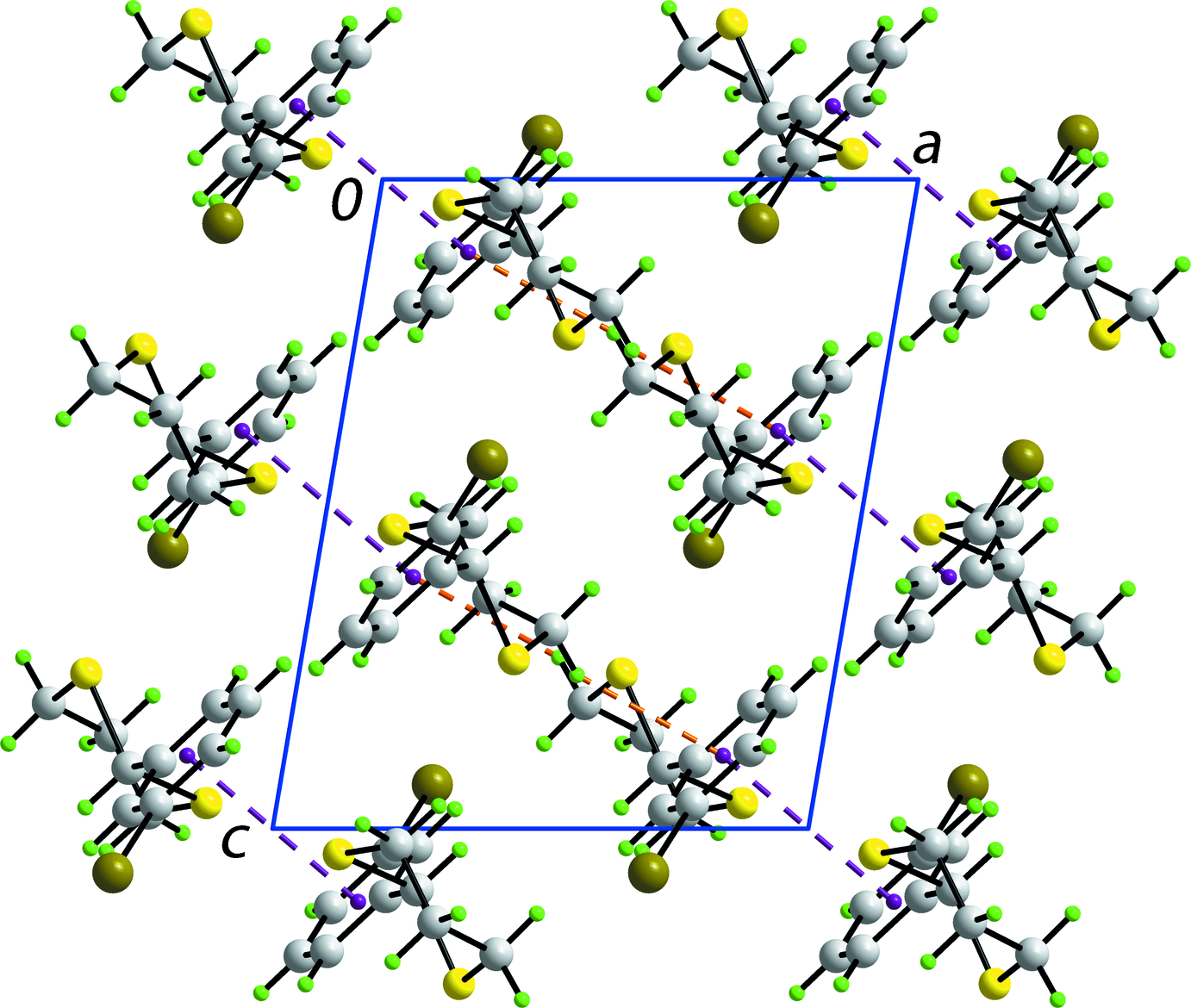

Supplement: Supplementary file 6 [file e-71-0o179-fig3.tif]
